# Transcriptomic responses of *Serratia liquefaciens* cells grown under simulated Martian conditions of low temperature, low pressure, and CO_2_-enriched anoxic atmosphere

**DOI:** 10.1038/s41598-018-33140-4

**Published:** 2018-10-08

**Authors:** Patricia Fajardo-Cavazos, Michael D. Morrison, Kathleen M. Miller, Andrew C. Schuerger, Wayne L. Nicholson

**Affiliations:** 1 Department of Microbiology and Cell Science, University of Florida, Merritt Island, FL 32953 USA; 2 Department of Plant Pathology, University of Florida, Merritt Island, FL 32953 USA

## Abstract

Results from previous experiments indicated that the Gram-negative α-proteobacterium *Serratia liquefaciens* strain ATCC 27592 was capable of growth under low temperature (0 °C), low pressure (0.7 kPa), and anoxic, CO_2_-dominated atmosphere–conditions intended to simulate the near-subsurface environment of Mars. To probe the response of its transcriptome to this extreme environment, *S. liquefaciens* ATCC 27592 was cultivated under 4 different environmental simulations: 0 °C, 0.7 kPa, CO_2_ atmosphere (Condition A); 0 °C, ~101.3 kPa, CO_2_ atmosphere (Condition B); 0 °C, ~101.3 kPa, ambient N_2_/O_2_ atmosphere (Condition C); and 30 °C, ~101.3 kPa, N_2_/O_2_ atmosphere (Condition D; ambient laboratory conditions). RNA-seq was performed on ribosomal RNA-depleted total RNA isolated from triplicate cultures grown under Conditions A-D and the datasets generated were subjected to transcriptome analyses. The data from Conditions A, B, or C were compared to laboratory Condition D. Significantly differentially expressed transcripts were identified belonging to a number of KEGG pathway categories. Up-regulated genes under all Conditions A, B, and C included those encoding transporters (ABC and PTS transporters); genes involved in translation (ribosomes and their biogenesis, biosynthesis of both tRNAs and aminoacyl-tRNAs); DNA repair and recombination; and non-coding RNAs. Genes down-regulated under all Conditions A, B, and C included: transporters (mostly ABC transporters); flagellar and motility proteins; genes involved in phenylalanine metabolism; transcription factors; and two-component systems. The results are discussed in the context of Mars astrobiology and planetary protection.

## Introduction

A central goal of Astrobiology is to understand the potential for habitability in the universe, including determination of the physical limits at which life can exist and the mechanisms used by living organisms to survive and grow in extreme environments^1,2^. Of particular interest have been investigations of whether Earth life could inhabit the environments of Mars, our closest potentially habitable neighbor. These studies are relevant to two related areas: (i) the potential for transport of life between Earth and Mars by natural impact processes (i.e., lithopanspermia), and (ii) the potential forward contamination of Mars as a consequence of human exploration activities (i.e., planetary protection)^3–7^. Due to their well-known resistance properties and ubiquitous distribution in extreme terrestrial environments, prokaryotes are considered the most likely candidates for interplanetary transfer by natural processes or human spaceflight activities, and much attention has logically focused on highly resistant or extremophilic microbes such as spores of *Bacillus subtilis* or the extremely radiation-resistant *Deinococcus radiodurans*^4,8^. Relatively less attention has been paid to more common environmental or human-associated bacteria which may actually be more likely to find themselves inadvertently transported to the martian surface. It is therefore important to understand the potential for terrestrial microbes not normally considered as extremophiles^9^ to proliferate in the martian environment, especially considering the recent increase of interest in travel to Mars by non-governmental entities not necessarily subject to the planetary protection requirements agreed to by the United Nations and administered by the Committee on Space Research (COSPAR)^10^.

Early studies confirmed that the UV radiation environment was a potent factor limiting the survival of Earth microbes on the martian surface, but that burial of cells at even minimal depths in the regolith provided effective UV shielding^3,11,12^. Subsequent studies testing the ability of various bacteria to grow under increasingly Mars-like laboratory simulations revealed that growth of most of the microbes tested, mostly strains obtained from laboratory collections or spacecraft assembly facilities, was inhibited by low temperature, low pressure, and anoxic, predominantly CO_2_ atmospheres, applied either singly or in pairwise combination^13,14^. Although most bacteria were unable to grow under the conditions applied in these early experiments, recently a small but growing subset of bacterial species have been discovered that can grow under simultaneously applied conditions of low temperature (0 °C), low pressure (0.7 kPa) and atmospheric gas composition (anoxic CO_2_) intended to simulate the physical conditions in the near subsurface of Mars. The origin of these bacteria were mostly Arctic soils or Siberian permafrost, and they were identified by 16S rDNA sequencing as hardy environmental species belonging to the genera *Bacillus, Carnobacterium, Clostridium, Cryobacterium, Exiguobacterium, Paenibacillus, Rhodococcus, Streptomyces*, and *Trichococcus*^15,16^.

Recently a screen of laboratory strains of bacteria for growth under the above “Mars-like” environmental conditions resulted in the identification of several species of the genus *Serratia* able to grow, including the type strain of *S. liquefaciens*, strain ATCC 27592^17^. Although in early publications the interest in *S. liquefaciens* stemmed from its being an opportunistic human pathogen^18–22^, recent reports point towards its physiological versatility allowing it to occupy ecologically diverse environments such as cold raw milk^23^, thawed cryoprecipitate^24^ or pulp mill effluent^25^. Because *Serratia* spp. have also been found on and within spacecraft and their assembly facilities^26,27^, *S. liquefaciens* is considered a potential forward contaminant of Mars-bound missions. The discovery that *S. liquefaciens* is capable of growth under Mars-like environmental conditions naturally leads to the question: what are the cellular and molecular mechanisms responsible? As a first step towards addressing this question, here we investigated how the global transcription profile (i.e., the transcriptome) of this organism responds when cultivated under environmental conditions mimicking those found in the martian near-subsurface. This study represents the first transcriptome profiling of a microorganism exposed to a simulation of the physical conditions prevailing on Mars.

## Results and Discussion

### Characterization of the *S. liquefaciens* transcriptomic response to different physical environments

**Figure 1:**
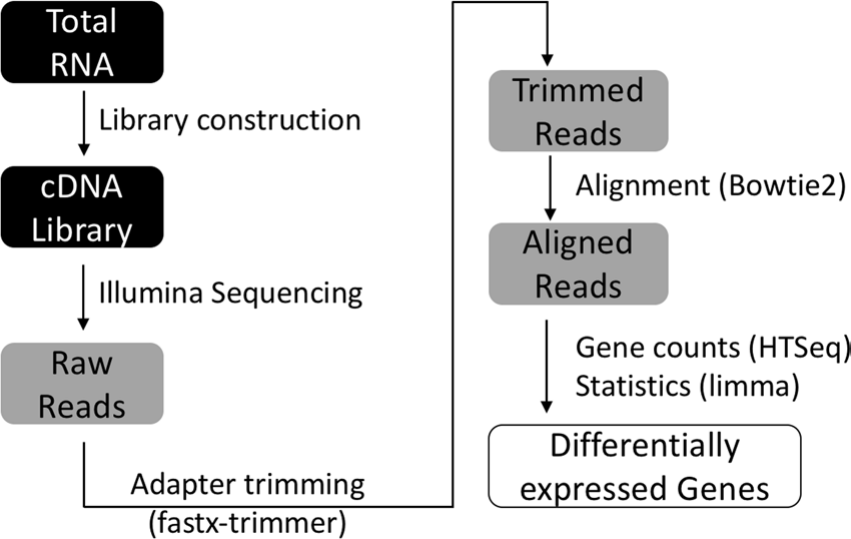
Bioinformatics work flow. Total RNA was isolated, and libraries were constructed and sequenced. Raw reads were filtered for quality and sequence contaminants and aligned to the reference genome of *S. liquefaciens* strain ATCC 27592 to calculate differential expression levels.

RNA-seq was utilized to analyze transcriptional changes in *S. liquefaciens* strain ATCC 27592 in response to four environmental conditions of temperature, pressure, and atmospheric gas composition simulating: the physical environment of Mars (Condition A), Earth-ambient laboratory conditions (Condition D), or a mixture of the two environments (Conditions B and C) (Table 1). Total RNA was isolated from cells grown under each condition as described in Materials and Methods, and determination of RNA integrity number (RIN) values (Table 1) demonstrated that all RNA samples were of the high quality required for further processing. RNA-seq analysis was performed on three replicates from each condition, resulting in 12 libraries which were sequenced on an Illumina HiSeq2500 instrument and subjected to the bioinformatic and statistical workflow described in detail in Materials and Methods and summarized in Fig. 1. Transcripts were defined as significantly differentially expressed if they exhibited a >4-fold difference with an adjusted *P* value < 0.01.

**Table 1:** Environmental conditions used in this study.

Condition	A	B	C	D
Temperature (°C)	0	0	0	30
Pressure (kPa)	0.7	~101.3	~101.3	~101.3
Gas Composition	CO_2_, anoxic	CO_2_, anoxic	80% N_2_/20% O_2_	80% N_2_/20% O_2_
Incubation time (days)	7	23	7	1
Number of TSA plates harvested per replicate	1	8	1	1
RIN values of replicates	9.6, 8.8, 8.8	9.4, 9.6, 8.9	9.5, 8.8, 9.3	9.0, 9.5, 9.2

### Overview of transcriptome analysis

**Figure 2:**
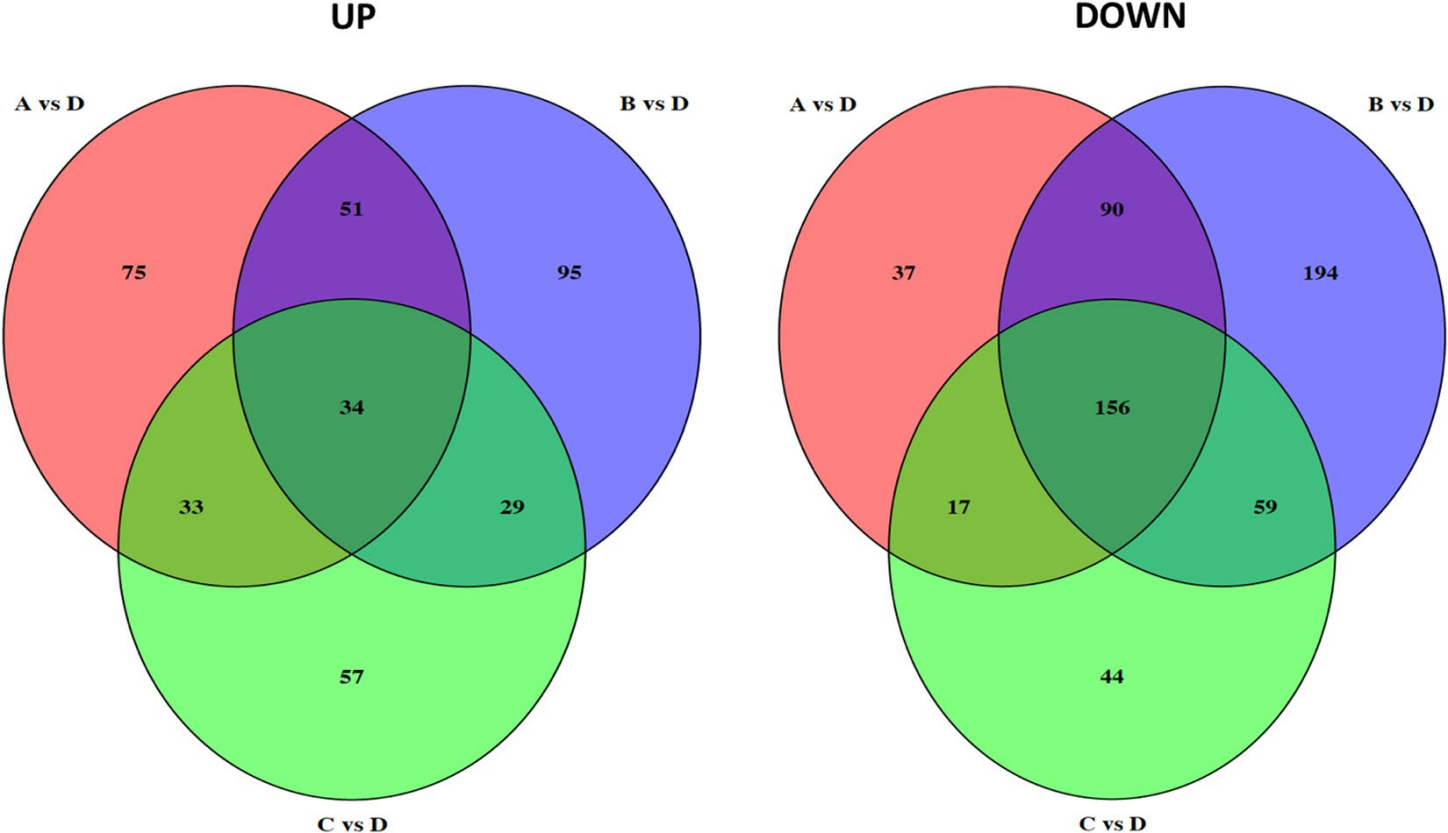
Venn diagram depicting the number of differentially up-regulated genes (left) and down-regulated genes (right) identified from Conditions A, B, and C compared to Condition D. Numbers in overlapping regions denote genes shared between the different comparisons and numbers in non-overlapping regions denote genes unique to each comparison. See Table 1 for description of the conditions.

In order to elucidate differences in gene expression of *S. liquefaciens* under the four conditions tested, each of the datasets obtained from Conditions A, B, or C were subjected to pairwise comparison with dataset D serving as the “Earth-like” control. The comparative analysis identified 493 differentially expressed genes in Condition A (193 up- and 300 down-regulated); 708 genes in condition B (209 up- and 499 down-regulated) and 429 genes in Condition C (153 up- and 276 down-regulated) with respect to the reference Condition D. The results of this analysis are summarized in Table 2 and represented graphically as Venn diagrams (Fig. 2). Inspection of the data revealed that exposure of *S. liquefaciens* to Conditions A, B, or C each produced its own set of transcriptome responses. These sets were partially unique and partially overlapping in any combination examined (Fig. 2). For example, a common set of 34 up-regulated and 156 down-regulated transcripts were found in cells exposed to all three Conditions A, B, and C; on the other hand, exposure to Condition A produced differential expression of 112 genes unique to Condition A (75 up- and 37 down-regulated) (Fig. 2). An Excel file listing the differential transcripts in common with Conditions A, B, and C vs. D is presented in Supplemental Table S1.

**Table 2:** Number of differentially expressed transcripts in each condition, compared to Condition D.

	A	B	C
Total	493	708	429
Up	193	209	153
Down	300	499	276

### Expression differences due to temperature

The transcriptome of *S. liquefaciens* ATCC 27592 consists of 5,003 total genes, of which 4,894 are protein-coding genes^28,29^. Comparison of Conditions C vs. D, in which only the temperature of incubation was different (0 °C vs. 30 °C), resulted in significant alteration of transcript levels of a total of 429 genes, or approximately 8.7% of the protein-coding transcriptome, with 153 and 276 transcripts being up- and down-regulated, respectively (Table 2). An Excel file listing the differentially expressed transcripts found in comparison of Condition C vs. D is presented in Supplemental Table S2.

### Expression differences due to temperature and atmospheric composition

Comparison of Conditions B vs. D, which differed both in temperature (0 °C vs. 30 °C) and atmospheric gas composition (anoxic CO_2_ vs. N_2_/O_2_), revealed differential expression of 708 total transcripts, or ~14.5% of the protein-coding transcriptome, with 209 and 499 transcripts being up- and down-regulated, respectively (Table 2). An Excel file listing the differentially expressed transcripts found in Condition B vs. D is presented in Supplemental Table S3. Clearly, exposure of *S. liquefaciens* to the combined conditions of low temperature and anoxic atmosphere provoked a massive restructuring of its transcriptome to accommodate growth under this new environmental regime.

### Expression differences in simulated Mars conditions

Cultures of *S. liquefaciens* grown under Conditions A vs. D differed by three parameters: the temperature of cultivation (0 °C vs. 30 °C), atmospheric gas composition (anoxic CO_2_ vs. N_2_/O_2_), and pressure (0.7 kPa vs. ~101 kPa); these represent a comparison of simulated Mars conditions vs. Earth (laboratory) conditions. Comparison of Condition A vs. D revealed a total of 493 transcripts altered, ~10.1% of the protein-coding transcriptome, with 193 and 300 transcripts being up- or down-regulated, respectively (Table 2). An Excel file listing the differentially expressed transcripts found in Condition A vs. D is presented in Supplemental Table S4.

We were surprised to find that Condition A (Mars simulation), which we presumed to be the most drastic deviation from Condition D (Earth control), resulted in a less dramatic restructuring of the *S. liquefaciens* transcriptome (10.1%) than was observed with the 0 °C, CO_2_-enriched anoxic atmosphere in Condition B (14.5%), and which was comparable in magnitude to the 0 °C only environment in Condition C (8.7%). It appeared as if the lowered pressure of Condition A was somehow ameliorating the dramatic transcriptomic response seen in Condition B. Similar effects among these conditions have been observed for the growth of *S. liquefaciens* and *Carnobacterium* spp.^14^,^15^ exposed to identical Mars-like conditions as were tested here. The observation that the low-pressure environment in Condition A seemed to mitigate some of the stresses in *S. liquefaciens* in Condition B prompted us to more closely examine gene expression differences due exclusively to pressure in Conditions A vs. B.

### Expression differences due to pressure alone (A vs. B)

Comparison of the transcriptome data from Condition A (0.7 kPa) vs. B (~101 kPa) revealed significant differential expression of 184 genes, or ~3.8% of the *S. liquefaciens* protein-coding genome, of which 178 were up-regulated and 6 were down-regulated under low pressure (Supplemental Table S5). Examination of the data sets failed to identify specific genes which might be reasoned to facilitate growth at low pressure. Indeed, the analysis revealed that among the most strongly up-regulated genes were those involved in transport and utilization of various sugars (lactose, arabinose, maltose, galactose, or general α-glucosides) or the polyol *m-*inositol. It should be noted that while TSA medium contains glucose at 0.25% final concentration, none of these other sugars are present in the medium. Of all the environmental parameters tested in our experiments, only low pressure is not encountered in nature on Earth, suggesting that up-regulation of this plethora of carbohydrate utilization systems at 0.7 kPa is likely maladaptive in *S. liquefaciens*. This may partly explain why *S. liquefaciens* grew so slowly and to such a low cell density when cultivated under Condition A (Table 1). Examination of the down-regulated genes showed that the most strongly affected were involved in transport of sulfate or the sulfur-containing amino acid cystine (Supplemental Table S5). Again, the possible relevance of the lowered expression of these genes to growth at low pressure would be speculative at best.

### Principal component analysis

**Figure 3:**
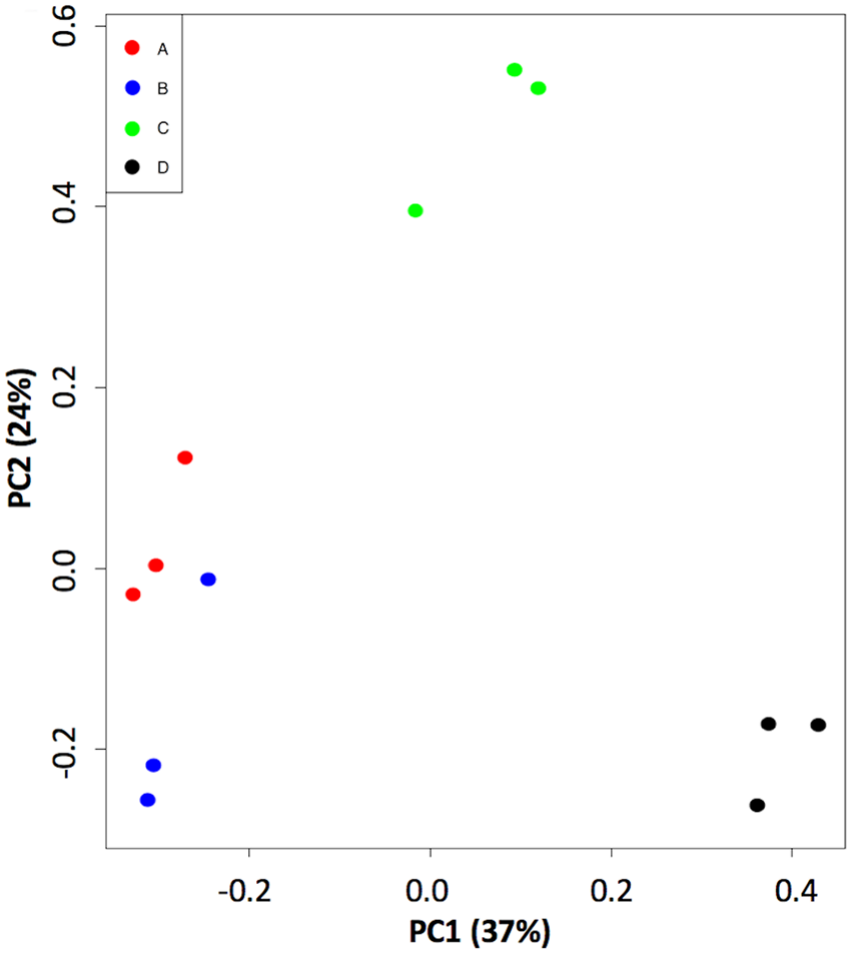
PCA plot of the data. Depicted are triplicate samples from Condition (**A**) (red) (**B**) (blue), (**C**) (green), and (**D**) (black). See Table 1 for description of the conditions.

Because the datasets generated from our RNA-seq experiments consisted of hundreds of genes, we turned to Principal Component Analysis (PCA) to assist us in assessing (i) the reproducibility of the replicates within a Condition, and (ii) whether different treatments result in different groupings. PCA was performed on the 12 datasets, and 4 distinct population clusters were identified which coincided with the 4 environmental conditions tested (Fig. 3). In the PCA, the first and second principal components explained 37% and 24% of the variance, respectively. In each population cluster, the three replicate samples grouped rather tightly, indicating good agreement among replicates for each condition. From inspection of the PCA plot it could be seen that Conditions A, B, and C differed markedly from Condition D, the laboratory control. However, Conditions A and B, which differed in pressure (0.7 kPa vs. ~101 kPa, respectively) clustered rather close to one another, indicating a relatively low degree of gene expression difference (Fig. 3).

### Assignment of KEGG categories

**Figure 4:**
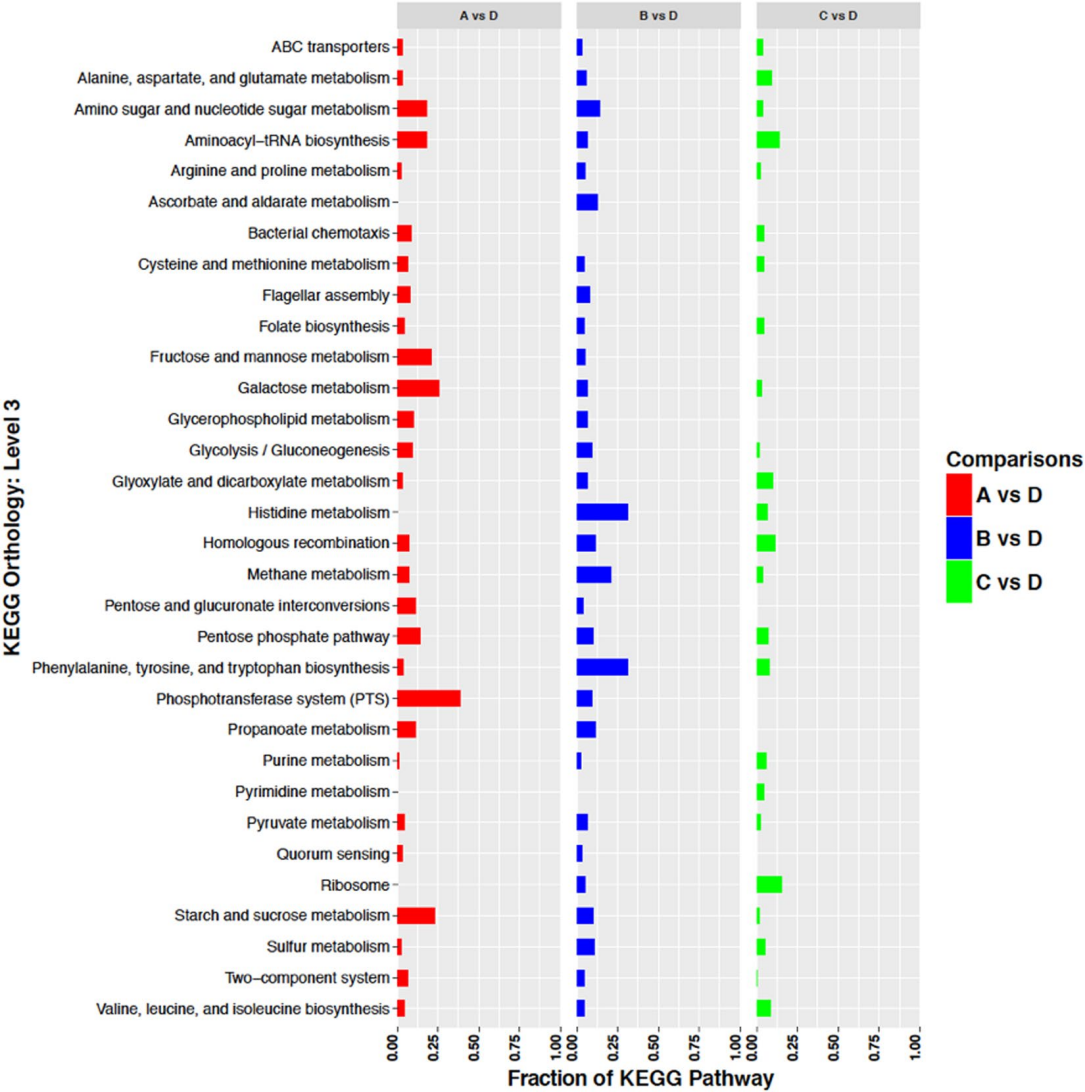
Up-regulated genes sorted by KEGG orthology. The number of genes of a particular KEGG pathway are depicted as a fraction of the total number of genes classified in that pathway in the genome of *S. liquefaciens*. See Table 1 for description of the conditions.

**Figure 5:**
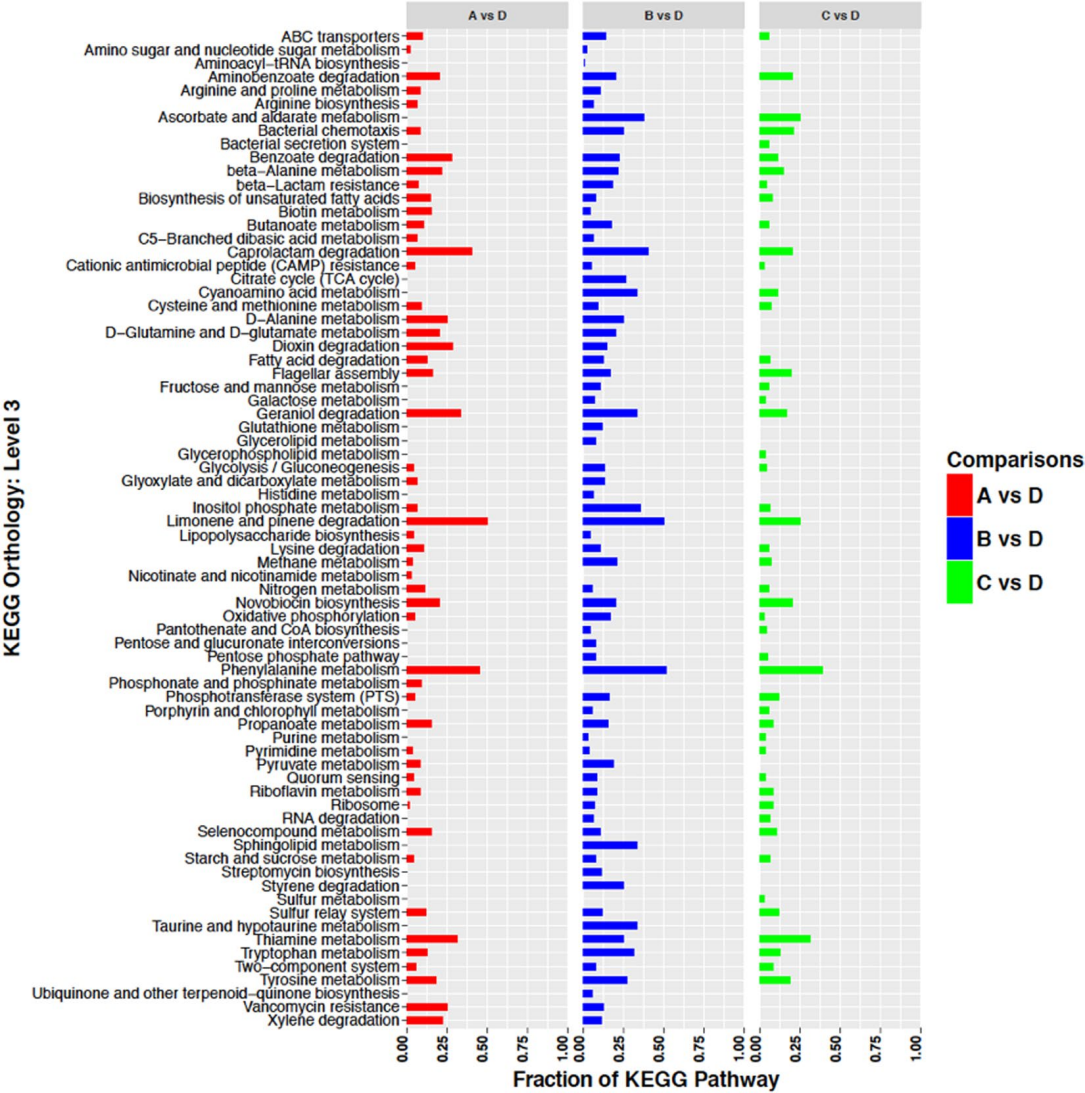
Down-regulated genes sorted by KEGG orthology. The number of genes of a particular KEGG pathway are depicted as a fraction of the total number of genes classified in that pathway in the genome of *S. liquefaciens*. See Table 1 for description of the conditions.

Genes whose transcripts were identified as significantly up- or down-regulated were further categorized and assigned to pathways as defined by the Kyoto Encyclopedia of Genes and Genomes (KEGG)^30^. Up- and down-regulated transcripts are presented in Figs 4 and 5 respectively. Visual inspection of the data revealed that genes up-regulated under all Conditions A, B, and C included: transporters (ABC and PTS transporters); genes involved in translation (ribosomes and their biogenesis, biosynthesis of both tRNAs and aminoacyl-tRNAs); DNA repair and recombination; and non-coding RNAs (Fig. 4). Genes down-regulated under all Conditions A, B, and C included: transporters (mostly ABC transporters); flagellar and motility proteins; genes involved in phenylalanine metabolism; transcription factors; and two-component systems (Fig. 5). Visual examination of the KEGG pathway data failed to identify specific functions that might be postulated to be responsible for the ability of *S. liquefaciens* to grow, or not, under a particular Condition.

### Adaptation of microbes to pressure changes

Of the environmental conditions used in this study, low temperature and anaerobic atmosphere can be found on Earth; only low pressure is a parameter unique to Mars, and the present study is the first to measure the transcriptomic response of a bacterium to low pressure comparable to that prevailing at the martian surface (0.7 kPa). To date, only one prior study has been published in which the transcriptomic response of a microorganism to low pressure exposure has been measured, that of the Gram-positive bacterium *Bacillus subtilis* grown at 5 kPa and 27 °C in Earth-normal 80% nitrogen/20% oxygen atmosphere^31^. In that study, exposure to low pressure led to up-regulation of transcripts from several global regulons (AbrB, CcpA, CodY, Fur, IolR, ResD, Rok, SigH, and Spo0A). Notably, the highest number of up-regulated genes, 86, belonged to the SigB-mediated General Stress Response (GSR) regulon^31^. In Gram-negative bacteria, the GSR is controlled by RNA polymerase containing the sigma factor RpoS^32^. In the present study, we did not observe significant up-regulation of expression of *rpoS* or RpoS-dependent GSR genes by *S. liquefaciens* exposed to simulated Mars conditions.

On the other end of the pressure spectrum, recent studies have reported the global transcriptional responses of piezophilic microorganisms to changes in pressure^33–35^. The closely related piezophiles *Desulfovibrio hydrothermalis* and *D. piezophilus* each grow at an optimum pressure of 10 MPa. Growth of the organisms at 0.1, 10, and 26 MPa resulted in alterations in expression of genes involved in lactate oxidation, energy metabolism, and amino acid biosynthesis^33,35^, but very few homologous genes were found to be differentially expressed in both organisms. Likewise, a study comparing the transcriptomic response of two closely related species, *Thermococcus barophilus* and *T. kodakarensis*, to changes in hydrostatic pressure failed to identify transcriptional responses in common^34^.

The previous studies cited, as well as the present study, were conducted with the intent of searching for new insights into the molecular mechanisms responsible for microbial adaptation to alterations in their pressure environment. In all cases, the sets of genes observed to be altered by pressure appeared to be organism-specific; no shared “pressure-responsive” genes or pathways have been discovered in the studies cited above. This finding parallels numerous studies which have tested explicitly for effects on the transcriptome of 2 or more environmental stressors acting simultaneously. Such studies have uncovered complex effects on gene expression that would not have been predicted from single stressor treatments. Furthermore, exposure to extreme environments can provoke maladaptive responses^36^. Because *S. liquefaciens*, like all other Earth organisms, has never been exposed to a low pressure of 0.7 kPa during its entire evolutionary history, it is not surprising that exposure to low pressure (Condition A vs. B), resulted in the up-regulation of a number of genes for utilization of carbohydrates not present in the medium, suggesting a maladaptive response to low pressure. What was surprising was that growth of *S. liquefaciens* at low pressure was not the result of a major rearrangement in its transcriptome, as visualized by the PCA plot (Fig. 3). This observation highlights the point that multiple interacting variables affect the transcriptome in ways that are neither intuitive nor predictable.

### Relevance to the potential growth of *S. liquefaciens* on Mars

The Martian surface is bathed in numerous biocidal factors among which UV irradiation, low water activity, low pressure, low temperature, and oxidizing conditions predominate^5,17^. Despite these harsh conditions, a few studies have demonstrated that as many as 31 bacterial species, including *S. liquefaciens*, are capable of active metabolism and growth under 0.7 kPa, 0 °C, and CO_2_-enriched anoxic conditions if cells are incubated in stable UV-protected, hydrated, and nutrient-rich environments^15–17^. The current study is the first to characterize up- and down-regulated transcripts at 0.7 kPa in a bacterium that may be plausibly transported to Mars (*Serratia* spp. have been recovered from spacecraft surfaces prior to launch^26,27^); thus our results begin to reveal how at least one terrestrial hypobarophile might be able to grow on Mars. The key for its success will be whether relevant metabolic pathways can remain active under 0.7 kPa, 0 °C, and CO_2_-dominated anoxic atmosphere, and whether cells can be dispersed into hydrated niches containing usable nutrients.

Although organic compounds have been directly detected^37^ or inferred^38^ in Martian regolith, the exact composition of the *in situ* organics remains unsolved because of the degradation of the organics by perchlorate salts during thermal pyrolysis of surface fines^38,39^. However, it is now accepted that *in situ* organics persist in the shallow subsurface on Mars. Furthermore, approx. 2.4 × 10^5^ kg of meteoritic carbon is accreted annually to Mars in the form of small carbonaceous chondrites and interplanetary dust particles (IDPs)^40^. The composition of carbonaceous chondrites^41^ and IDPs^42^ have been extensively studied, and a comparison between accreted organics to Mars and the up-regulated pathways for *S. liquefaciens* described here for 0.7 kPa Mars simulations suggest that at least some common metabolic pathways may remain active on the surface of Mars in which accreted organics might be accessible. For example, KEGG analyses indicated that several amino acid, purine, and sugar carbohydrate pathways were up-regulated at 0.7 kPa (Fig. 4), suggesting that these metabolic pathways might remain active on Mars, and that cells of *S. liquefaciens* might be able to utilize similar classes of organics (i.e., aldehydes, amino acids, ketones, and purines) found in IDPs and carbonaceous chondrites^41–43^. Our results are consistent with the hypothesis that some terrestrial bacteria are capable of metabolism and growth in stable UV-protected and hydrated niches on Mars, and that accreted or *in situ* organics may provide the required nutrients for heterotrophic metabolism. However, more research is required to examine the malleability of the transcriptome of *S. liquefaciens* and other hypobarophiles to fluctuating conditions on the Martian surface.

Finally, the habitability of any martian landing site will be a key consideration for future robotic and crewed spacecraft in which either life-detection experiments will be conducted or human exploration traverses completed. If: (i) hypobarophilic microorganisms are present on spacecraft or payload surfaces prior to launch; (ii) landing sites are near terrains that might harbor an extant Mars microbiota or support the growth of terrestrial microbes (i.e., defined as Special Regions^5^); and (iii) metabolic pathways remain active at 0.7 kPa that can utilize *in situ* or accreted organics on Mars, then the risk of the forward contamination of such sites will remain high.

## Materials and Methods

### Bacterial strain and medium

The bacterial strain used in this study was *Serratia liquefaciens* strain ATCC 27592 obtained from the American Type Culture Collection, Manassas, VA USA. Its complete genome sequence has been determined^28^ and is deposited in the National Center for Biotechnology Information (NCBI) GenBank database under accession number CP006252.1. Medium used throughout was Tryptic Soy Agar (TSA) (BD Difco, Franklin Lakes, NJ USA).

### Growth conditions

The experimental conditions used are described in Table 1. In brief, simulated Mars conditions (Condition A) were chosen based on the near-ubiquitous extent of low pressure between 0.7 and 1.0 kPa at the surface^44,45^, the nominal CO_2_-dominated (96%) atmosphere^46^, and the requirement to maintain a stable liquid environment near the triple-point of water at 0 °C and 0.7 kPa^47^. The control conditions B, C, and D were chosen in order to discriminate the effects of pressure, gas composition, and temperature, respectively, compared to the Martian conditions tested. Although numerous other environmental and geochemical conditions might be present at the surface of Mars (e.g., diel temperature swings, low water activity, UV irradiation, high salt or perchlorate concentrations, oligotrophic nutrient regimes in the regolith) the first-order Mars simulations used here were chosen to create a stable hydrated and nutrient-rich environment in order to characterize transcriptomic changes in *S. liquefaciens* under a core set of three common environmental conditions on the Martian surface. The simulated Martian conditions used are consistent with models that suggests stable liquid water may occur on present-day Mars^47,48^.

Cultures were prepared in triplicate. For each replicate at each growth condition, the number of TSA plates indicated in Table 1 was inoculated with cells from a freshly-prepared culture grown overnight in a laboratory incubator at 30 °C. Cultures were incubated in a desiccator/low-pressure control system as described previously^16,17^. Briefly, TSA plates seeded with *S. liquefaciens* cells were placed into a 4-L polycarbonate desiccator (model 08-642-7, Fisher Scientific, Pittsburg, PA, USA), surrounded by four anaerobic pouches (Remel Anaerobic Pack sachets, Fisher Scientific), the desiccator top attached to the desiccator body, and the lab atmosphere flushed with filter-sterilized, ultra-high purity CO_2_ gas for 3–4 min, resulting in an atmosphere of essentially 100% CO_2_. The desiccator was sealed, connected to an externally mounted pump and low-pressure control system (model PU-842, KNF Neuberger, Trenton, NJ, USA), and placed within a microbial incubator set at 0 °C.

The equilibration process to stabilize the cultures at low-pressure conditions without boiling the media required approximately 60 min. The desiccators were pumped down to 10, 5, 2.5, and 0.7 kPa sequentially for approx. 15 min per time-step. The cumulative 60-min equilibration time allowed the cultures to outgas laboratory air or dissolved CO_2_ while also simultaneously cooling the media slowly to 0 °C. At 7-day intervals, the desiccators were vented to lab air and exhausted anaerobic pouches were replaced with fresh ones. Anaerobic pouches similar to those described above have been shown to remove O_2_ to a concentration of ≤0.1% within 1 h in small closed containers^49^; this concentration closely matches the concentration of O_2_ in the martian atmosphere (0.145%)^46^.

After incubation, cells were removed from agar surfaces by rubbing with a sterile glass rod and suspended in sterile, ice-cold PBS buffer^50^. Cell suspensions were immediately centrifuged (7,000 × g, 0 °C, 20 min) and the cell pellets stored at −70 °C until use.

### Isolation of total RNA

Total RNA was extracted from cell pellets and treated with RNase-free DNase I using the RiboPure^TM^ RNA Purification Kit (Thermo Fisher Scientific, Waltham, MA) following the manufacture’s protocol. Total RNA content in samples was determined using a Qubit fluorometer (Invitrogen, Thermo Fisher Scientific Inc, Waltham, MA). Sample quality was measured using the RNA 6000 Nano Chip and Agilent 2100 Bioanalyzer (Agilent Technologies, Santa Clara, CA). The RNA Integrity Number (RIN)^51^ values of the samples are presented in Table 1.

### RNA-seq and data analysis

RNA samples were shipped on dry ice to the Hudson Alpha Institute for Biotechnology, (Huntsville, AL) where they were subjected to ribosomal RNA reduction, library preparation, and multiplex 50-nucleotide single-end sequencing on an Illumina HiSeq2500 instrument. The raw Illumina sequences were imported into the University of Florida’s High Performance Research Computer HiPerGator 2 platform as.fastq files for further processing. Low-quality base calls were trimmed from the sequences using fastx-trimmer version 0.0.14 with a quality threshold of 33. Trimmed reads were mapped to the *S. liquefaciens* strain ATCC 27592 genome (NCBI RefSeq NC_021741.1)^28^ using bowtie2 version 2.3.2^52^, and the aligned sequence files were imported into HTSeq version 0.6.1^53^ for read counting. All differential expression analyses were performed in R version 3.4.2 using the package *limma*^54–56^. Per *limma* recommendation, transcripts with less than 10 total counts across all samples were removed. Gene counts were normalized using the trimmed mean of M values (TMM) method, and the normalized counts were transformed using the built-in voom method. The log_2_-fold change values for each transcript were determined using *limma*’s default eBayes method and the *P* values were adjusted for multiple testing using the Benjamini-Hochberg method^57^. Genes were considered to be differentially expressed if they exhibited at least a 4-fold (i.e., a log_2_ value >2) difference in transcript levels between conditions with a Benjamini-Hochberg adjusted *P* value less than 0.01. Principal Component Analysis (PCA) was performed on the normalized gene counts using the built-in R package *stats*, and the loading scores for the first two principal components were plotted in R. The processed read data are available in the National Center for Biotechnology Information (NCBI) Gene Expression Omnibus (GEO) database under accession number GSE120390.

Functional enrichment analysis was performed using BLAST2GO version 4.1.9^58^. FASTA sequences for differentially expressed genes were retrieved from the PATRIC database^59^ using the KEGG locus tags associated with each gene. A BlastX-fast program was run for all FASTA sequences against the non-redundant database with an α-proteobacterial (taxa: 28211, Alphaproteobacteria) filter. Default mapping and annotation parameters were used, and Blast expectation values were set at a threshold of 1.0E-3. Annotation configurations were run with the default parameters and successfully annotated hits were mapped to Gene Ontology (GO) terms and pathways in the KEGG database^30,60,61^.

## Electronic supplementary material

S1

S2

S3

S4

S5

## References

[1] Chyba, C. F. & Hand, K. P. In *Annu. Rev. Astron. Astrophys*. Vol. 43 31–74. (Annual Reviews, 2005).

[2] Cockell CS (2016). Habitability: A Review. Astrobiology.

[3] Fajardo-Cavazos P, Schuerger AC, Nicholson WL (2007). Testing interplanetary transfer of bacteria between Earth and Mars as a result of natural impact phenomena and human spaceflight activities. Acta Astronaut..

[4] Nicholson WL, Munakata N, Horneck G, Melosh HJ, Setlow P (2000). Resistance of *Bacillus* endospores to extreme terrestrial and extraterrestrial environments. Microbiol. Mol. Biol. Rev..

[5] Rummel JD (2014). A new analysis of Mars “Special Regions”: findings of the second MEPAG Special Regions Science Analysis Group (SR-SAG2). Astrobiology.

[6] Nicholson WL (2009). Ancient micronauts: interplanetary transport of microbes by cosmic impacts. Trends Microbiol..

[7] Nicholson WL, Schuerger AC, Race MS (2009). Migrating microbes and planetary protection. Trends Microbiol..

[8] Munteanu A, Uivarosi V, Andries A (2015). Recent progress in understanding the molecular mechanisms of radioresistance in *Deinococcus* bacteria. Extremophiles.

[9] Horikoshi, K. *et al*. *Extremophiles Handbook*. 608 pp. (Springer, 2011).

[10] Meltzer, M. *When Biospheres Collide: A History of NASA’s Planetary Protection Programs*. 521 pp. (NASA, Washington DC, 2011).

[11] Nicholson WL, Schuerger AC, Setlow P (2005). The solar UV environment and bacterial spore UV resistance: considerations for Earth-to-Mars transport by natural processes and human spaceflight. Mutat. Res..

[12] Moores JE, Smith PH, Tanner R, Schuerger AC, Venkateswaran KJ (2007). The shielding effect of small-scale martian surface geometry on ultraviolet flux. Icarus.

[13] Schuerger AC, Nicholson WL (2006). Interactive effects of hypobaria, low temperature, and CO_2_ atmospheres inhibit the growth of mesophilic *Bacillus* spp. under simulated martian conditions. Icarus.

[14] Berry BJ, Jenkins DG, Schuerger AC (2010). Effects of simulated Mars conditions on the survival and growth of *Escherichia coli* and *Serratia liquefaciens*. Appl Environ Microbiol.

[15] Nicholson WL, Krivushin K, Gilichinsky D, Schuerger AC (2013). Growth of *Carnobacterium* spp. from permafrost under low pressure, temperature, and anoxic atmosphere has implications for Earth microbes on Mars. Proc Natl Acad Sci USA.

[16] Schuerger AC, Nicholson WL (2016). Twenty species of hypobarophilic bacteria recovered from diverse soils exhibit growth under simulated martian conditions at 0.7 kPa. Astrobiology.

[17] Schuerger AC, Ulrich R, Berry BJ, Nicholson WL (2013). Growth of *Serratia liquefaciens* under 7 mbar, 0 °C, and CO_2_-enriched anoxic atmospheres. Astrobiology.

[18] Grohskopf LA (2001). *Serratia liquefaciens* bloodstream infections from contamination of epoetin alfa at a hemodialysis center. N Engl J Med.

[19] Engelhart S (2003). Severe *Serratia liquefaciens* sepsis following vitamin C infusion treatment by a naturopathic practitioner. J Clin Microbiol.

[20] Mossad SB (2000). The world’s first case of *Serratia liquefaciens* intravascular catheter-related suppurative thrombophlebitis and native valve endocarditis. Clin Microbiol Infect.

[21] Pinna A, Usai D, Sechi LA, Carta A, Zanetti S (2011). Detection of virulence factors in *Serratia* strains isolated from contact lens-associated corneal ulcers. Acta Ophthalmol.

[22] Roth VR (2000). Transfusion-related sepsis due to *Serratia liquefaciens* in the United States. Transfusion.

[23] Machado SG (2015). *Pseudomonas* spp. and *Serratia liquefaciens* as predominant spoilers in cold raw milk. J Food Sci.

[24] Ramirez-Arcos S, Jenkins C, Sheffield WP (2017). Bacteria can proliferate in thawed cryoprecipitate stored at room temperature for longer than 4 h. Vox Sang.

[25] Haq I, Kumar S, Kumari V, Singh SK, Raj A (2016). Evaluation of bioremediation potentiality of ligninolytic *Serratia liquefaciens* for detoxification of pulp and paper mill effluent. J Hazard Mater.

[26] Ghosh S, Osman S, Vaishampayan P, Venkateswaran K (2010). Recurrent isolation of extremotolerant bacteria from the clean room where Phoenix spacecraft components were assembled. Astrobiology.

[27] Moissl C (2007). Molecular bacterial community analysis of clean rooms where spacecraft are assembled. FEMS Microbiol Ecol.

[28] Nicholson WL (2013). Complete Genome Sequence of *Serratia liquefaciens* Strain ATCC 27592. Genome Announc.

[29] Markowitz VM (2012). IMG: the integrated microbial genomes database and comparative analysis system. Nucleic Acids Research.

[30] Kanehisa M, Goto S (2000). KEGG: kyoto encyclopedia of genes and genomes. Nucleic Acids Res.

[31] Waters SM, Robles-Martínez JA, Nicholson WL (2014). Exposure of *Bacillus subtilis* to low pressure (5 kPa) induces several global regulons including the *sigB*-mediated General Stress Response. Appl Environ Microbiol.

[32] Landini P, Egli T, Wolf J, Lacour S (2014). sigmaS, a major player in the response to environmental stresses in *Escherichia coli:* role, regulation and mechanisms of promoter recognition. Environmental Microbiology Reports.

[33] Amrani A (2016). Deciphering the adaptation strategies of *Desulfovibrio piezophilus* to hydrostatic pressure through metabolic and transcriptional analyses. Environ Microbiol Rep.

[34] Vannier P, Michoud G, Oger P, Marteinsson V, Jebbar M (2015). Genome expression of *Thermococcus barophilus* and *Thermococcus kodakarensis* in response to different hydrostatic pressure conditions. Res Microbiol.

[35] Amrani A (2014). Transcriptomics reveal several gene expression patterns in the piezophile *Desulfovibrio hydrothermalis* in response to hydrostatic pressure. PLoS One.

[36] DeBiasse MB, Kelly MW (2016). Plastic and evolved responses to global change: what can we learn from comparative transcriptomics?. J Hered.

[37] Ming DW (2014). Volatile and organic compositions of sedimentary rocks in Yellowknife Bay, Gale crater, Mars. Science.

[38] Navarro-Gonzalez R, Vargas E, de la Rosa J, Raga AC, McKay CP (2010). Reanalysis of the Viking results suggests perchlorate and organics at midlatitudes on Mars. Journal of Geophysical Research-Planets.

[39] Glavin DP (2013). Evidence for perchlorates and the origin of chlorinated hydrocarbons detected by SAM at the Rocknest aeolian deposit in Gale Crater. Journal of Geophysical Research-Planets.

[40] Flynn GJ (1996). The delivery of organic matter from asteroids and comets to the early surface of Mars. Earth, Moon & Planets.

[41] Sephton MA (2002). Organic compounds in carbonaceous meteorites. Nat. Prod. Rep..

[42] Sephton MA, Botta O (2005). Recognizing life in the Solar System: guidance from meteoritic organic matter. Int. J. Astrobiol..

[43] Clemett SJ, Maechling CR, Zare RN, Swan PD, Walker RM (1993). Identification of complex aromatic molecules in individual interplanetary dust particles. Science.

[44] Gómez-Elvira J (2014). Curiosity’s rover environmental monitoring station: Overview of the first 100 sols. J. Geophys. Res. Planets.

[45] Hess SL, Ryan JA, Tillman JE, Henry RM, Leovy CB (1980). The annual cycle of pressure on Mars measured by Viking Landers 1 and 2. Geophys. Res. Letters.

[46] Mahaffy PR (2013). Abundance and isotopic composition of gases in the martian atmosphere from the Curiosity rover. Science.

[47] Haberle RM (2001). On the possibility of liquid water on present-day Mars. J. Geophys. Res..

[48] Lobitz B, Wood BL, Averner MM, McKay CP (2001). Use of spacecraft data to derive regions on Mars where liquid water would be stable. Proc Natl Acad Sci USA.

[49] Van Horn KG, Warren K, Baccaglini EJ (1997). Evaluation of the AnaeroPack system for growth of anaerobic bacteria. J. Clin. Microbiol..

[50] Nicholson, W. L. & Setlow, P. In *Molecular biological methods forBacillus*. (eds C. R. Harwood & S. M. Cutting) 391–450 (J. Wiley & Sons, 1990).

[51] Schroeder A (2006). The RIN: an RNA integrity number for assigning integrity values to RNA measurements. BMC Mol Biol.

[52] Langmead B, Trapnell C, Pop M, Salzberg SL (2009). Ultrafast and memory-efficient alignment of short DNA sequences to the human genome. Genome Biol.

[53] Anders S, Pyl PT, Huber W (2015). HTSeq–a Python framework to work with high-throughput sequencing data. Bioinformatics.

[54] Ritchie ME (2015). limma powers differential expression analyses for RNA-sequencing and microarray studies. Nucleic Acids Res.

[55] Law CW, Chen Y, Shi W, Smyth GK (2014). Voom: precision weights unlock linear model analysis tools for RNA-seq read counts. Genome Biol.

[56] Liu R (2015). Why weight? Modelling sample and observational level variability improves power in RNA-seq analyses. Nucleic Acids Res.

[57] Benjamini Y, Hochberg Y (1995). Controlling the false discovery rate: a practical and powerful approach to multiple testing. J. Royal Statist. Soc. B.

[58] Götz S (2008). High-throughput functional annotation and data mining with the Blast2GO suite. Nucleic Acids Res.

[59] Wattam AR (2018). Assembly, annotation, and comparative genomics in PATRIC, the All Bacterial Bioinformatics Resource Center. Methods Mol Biol.

[60] Kanehisa M, Sato Y, Kawashima M, Furumichi M, Tanabe M (2016). KEGG as a reference resource for gene and protein annotation. Nucleic Acids Res.

[61] Kanehisa M, Furumichi M, Tanabe M, Sato Y, Morishima K (2017). KEGG: new perspectives on genomes, pathways, diseases and drugs. Nucleic Acids Res.

